# Fixation stability comparison of bone screws based on thread design: buttress thread, triangle thread, and square thread

**DOI:** 10.1186/s12891-022-05751-6

**Published:** 2022-08-30

**Authors:** Xiaoreng Feng, Zhaopei Luo, Yupeng Li, Yiyi Yao, Weichen Qi, Bin Chen, Hongfeng Liang

**Affiliations:** 1 Department of Orthopaedics and Traumatology, Yangjiang People’s Hospital, No. 42 Dongshan Road, Jiangcheng District, 529500 Yangjiang, China; 2 Division of Orthopaedics and Traumatology, Department of Orthopaedics, Nanfang Hospital, Southern Medical University, No. 1838 North Guangzhou Avenue, Guangzhou, 510515 China; 3 Department of Orthopaedics and Traumatology, Li Ka Shing Faculty of Medicine, the University of Hong Kong, Hong Kong, China

**Keywords:** Bone screw, Buttress thread, Triangle thread, Square thread, Fixation stability

## Abstract

**Background:**

The influence of thread profile on the fixation stability of bone screws remains unclear. This study aimed to compare the fixation stability of screws with different thread profiles under several loading conditions.

**Methods:**

Bone screws that differed in thread profile (buttress, triangle, and square thread) only were made of stainless steel. Their fixation stabilities were evaluated individually by the axial pullout test and lateral migration test, besides, they were also evaluated in pairs together with a dynamic compression plate and a locking plate in polyurethane foam blocks under cyclic craniocaudal and torsional loadings.

**Results:**

The triangle-threaded and square-threaded screws had the highest pullout forces and lateral migration resistance. When being applied to a dynamic compression plate, higher forces and more cycles were required for both triangle- and square-threaded screws to reach the same displacement under cyclic craniocaudal loading. On the other hand, the triangle-threaded screws required a higher torque and more cycles to reach the same angular displacement under cyclic torsional loading. When being applied to a locking plate, the square-threaded screws needed higher load, torque, and more cycles to reach the same displacement under both cyclic craniocaudal and torsion loadings.

**Conclusions:**

The triangle-threaded screws had superior pullout strength, while square-threaded screws demonstrated the highest lateral migration resistance. Moreover, dynamic compression plate fixation with triangle- and square-threaded screws achieved more favorable fixation stability under craniocaudal loading, while triangle-threaded screws demonstrated superior fixation stability under torsional loading. Locking plate fixation with a square-threaded screw achieved better fixation stability under both loading types.

## Background

Bone screw, a powerful mechanical device with versatile functions, has been frequently applied in orthopedic surgeries [1]. It can be used alone to repair simple fractures or in combination with a plate or rod to repair more complicated fractures [1].

Even with good clinical outcomes, the screw failure rate reported clinically is high, especially among patients with poor bone quality [2–7]. The intertrochanteric fracture screw failure rate varies from 3 to 5% [2, 3], the proximal humeral fracture screw failure rate is between 15 and 40% [4, 5], while the pedicle screw failure rate in multilevel fusion for degenerative diseases of the lumbar spine can be up to 50% [6, 7]. It has been suggested that at least one million pedicle screw failures occur annually worldwide due to loosening and/or migration. Some failures may not have significant clinical consequences, but some may require immediate and costly surgical revision [7].

Improving the fixation strength of bone screws is a feasible way to reduce the failure rate [8]. Screw thread is the determinant component that governs the initial screw stability of fixation, therefore, it is extremely important to choose the right thread profile for the bone screw [9–11]. Bone screws are derived from industrial screws with various thread designs for different purposes [12]. Generally speaking, triangle thread is used as a fastener, while the trapezoidal, square, and buttress threads are utilized as power screws. Consequently, when bone screws were first used in orthopedic surgery, they were designed as the triangle threads for connecting bone. In the 1940s, Belgian surgeon Robert Danis proposed three key screw design features tailored to the human bone: a ratio of the exterior diameter to the core diameter of 3:2, compared with that of 4:3 for a typical industrial metal screw; a reduction in the thread surface area to 1/6 of that of an industrial metal screw since the human bone has approximately 1/6 of the strength of metal, and a buttress thread design to replace the standard triangle thread for its greater axial holding power [13, 14]. Since then, buttress threads have become the standard thread profile for bone screws.

The thread design of a bone screw can vary in pitch, depth, and shape. The relationships between the mechanical properties of bone screws and the modifications of thread pitch and depth have been well documented [15]. The ratio of thread depth to pitch has been expressed as a percentage (the so-called “thread-shape factor”), with the smaller pitch and greater depth demonstrating the greater pullout strength in porous synthetic materials and thus the higher primary stability [15]. The influence of different thread profiles on the pullout strength of bone screws has been evaluated in several studies [16, 17]; however, no existing studies have focused on the other loading conditions of bone screws with different thread profile designs that are more relevant to the physiological loading of human body. Lateral migration resistance, the ability to resist lateral movements of the screw during physiological loading of the bone-implant construct, has been proved to be associated with the fixation stability of bone screws [18, 19]. Numerous studies and even ASTM standards have already proposed loading conditions resulting in lateral migration of the screws. Nonetheless, these studies are not specifically meant for screw characterization [20, 21]. Therefore, this study aimed to reveal and compare the fixation stability of screws with buttress, triangle, and square threads in synthetic bones under different loading conditions. To be specific, screws with different thread designs were tested individually by the axial pullout test and lateral migration test, besides, they were tested in pairs together with a dynamic compression plate (DCP) and a locking plate (LP) under cyclic craniocaudal and torsional loadings.

## Methods

Locking screws and compression screws of 3 different thread designs (buttress thread, square thread, and triangle thread) were manufactured from the 316 LVM stainless steel. All the screws were self-tapping screws with a thread pitch of 2.0 mm, major and minor diameters of 4.5 mm and 3.2 mm, respectively, screw shaft length of 38 mm, and had the same cutting flute design. The 4.5-mm screws were used for compatibility with the standard instrumentation for the 4.5-mm AO cortical screws by relying on the identical drill bits (diameter, 3.2 mm). All the screws were used once in the following tests.

**Fig. 1:**
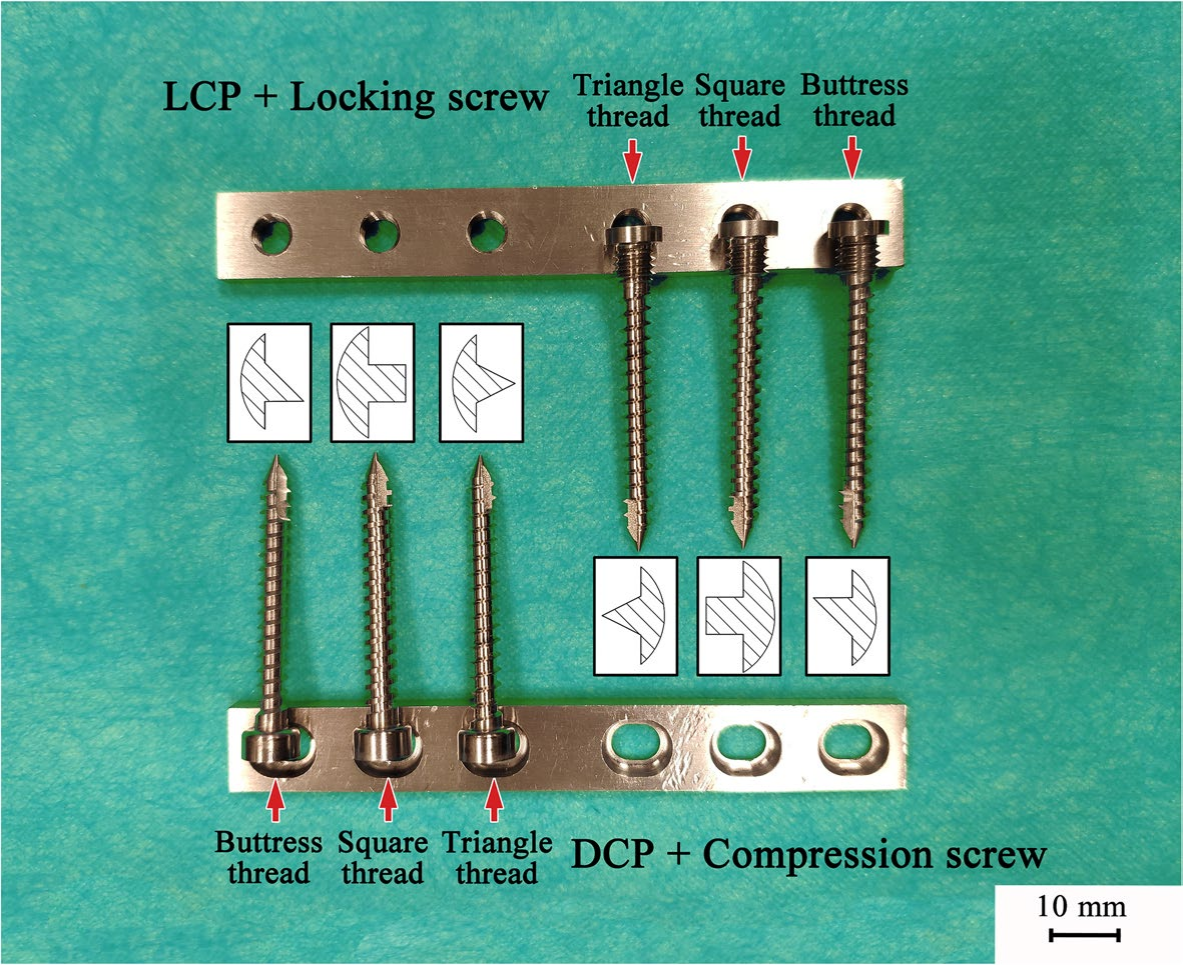
Screws and corresponding plates for biomechanical tests in this study

LP and DCP were specially designed for the above locking and compression screws and manufactured from the 316 LVM stainless steel (Fig. 1). Both plate types were 12 mm wide, 95 mm long, 4 mm thick, with a hole-spacing of 15 mm, which represented the plates used for human long bone fracture repair. All the plates were utilized once in the following tests.

Solid rigid polyurethane foam blocks with a density of 0.32 g/cm^3^ (Sawbones, Vashon, Washington, USA) that adhered to ASTM F-1839-08 were used [22]. The density was chosen because it was within the range of densities for normal cancellous bone and was validated by screw pullout testing in previous studies [23–25].

Thereafter, individual screw axial pullout and lateral migration tests, and multi-screws stability of plate fixation test, which were based on our previously published work [19], were conducted to evaluate bone screws with different thread designs.

### Individual screw axial pullout test

**Fig. 2:**
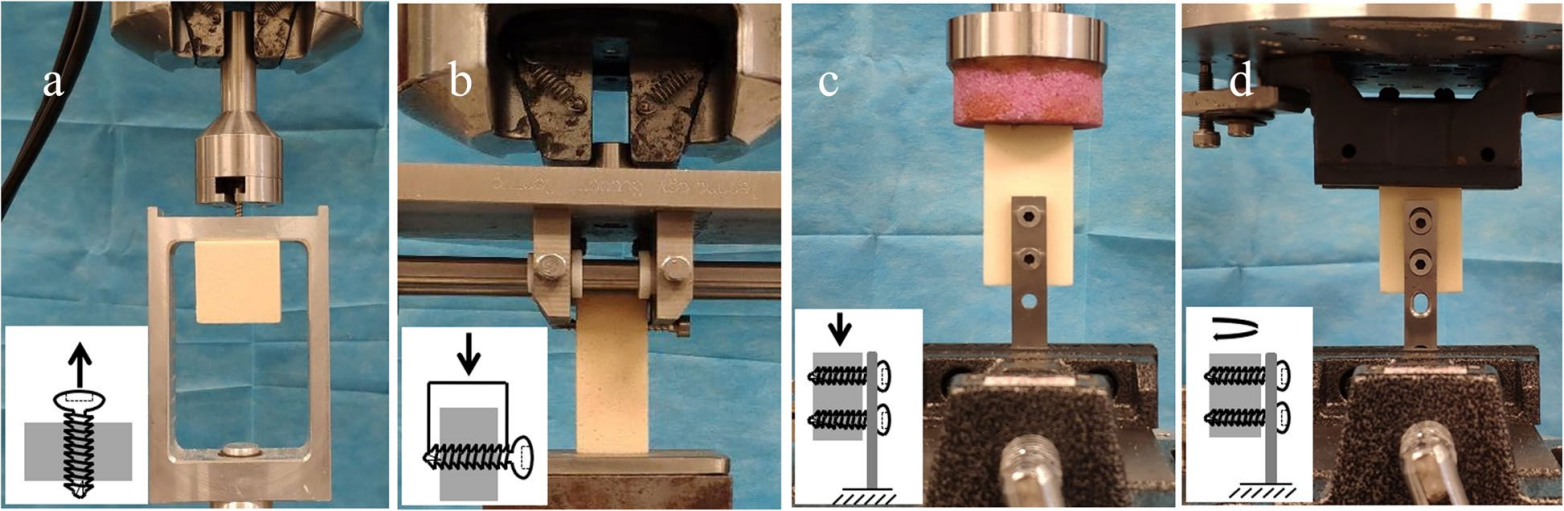
Experimental setups used in this study. **a** Individual screw axial pullout test; **b** individual screw lateral migration test; **c**, **d** multi-screw stability of plate fixation test under craniocaudal loading **c**) and torsional loading **d**)

In brief, polyurethane foam blocks were cut into small pieces (size, 40 × 40 × 40 mm) (Fig. 2 a). Thereafter, a pilot hole was made all the way through the center of each polyurethane foam block by using a 3.2-mm drill bit. The blocks were later divided into three groups for use with the three different thread types. Five screws per group were screwed 25 mm deep into the pre-drilled pilot holes, in other words, a 25-mm long section of thread was anchored vertically into the foam block. Each screw/block construct was thus mounted onto the load cell (0–1000 N) of an MTS 858 Mini Bionix (MTS, Inc., Minnesota, USA) hydraulic loading machine with a custom-made jig, so as to ensure strict axial tension on the screw. In line with previous standards [26], the screws were extracted 10 mm from their starting point under displacement control at a rate of 5 mm/min.

### Individual screw lateral migration test

The polyurethane foam blocks were cut into small pieces (size, 20 × 20 × h50 mm) (Fig. 2 b). Afterward, a 20-mm long pilot hole was drilled all the way through each polyurethane foam block along one of the 20-mm axes by using a 3.2-mm drill bit. Five screws per group were screwed into the pilot hole until the cutting flute exited the foam block. Later, a 20-mm long section of thread was anchored horizontally into the foam block for all screws. The foam block was fixed while the two ends of the screw were compressed vertically at 5 mm/min to a 10-mm displacement using an MTS 858 Mini Bionix (MTS, Inc., Minnesota, USA) hydraulic loading machine.

The force versus displacement data were collected at 10 Hz as the screw was pulled out axially or migrated laterally through the foam block. Subsequently, a force-displacement curve was plotted, and the stiffness, yield force, and maximum force of the curve were calculated. To be specific, stiffness was calculated as the slope of the best-fit line for the linear region of the force-displacement curve. The yield force was determined using a 0.015-mm offset parallel to the stiffness [27]. Moreover, the maximum force for the axial pullout test was the force measured at the peak of the curve. For the lateral migration test, it was defined as the force measured at 5 mm of lateral migration. Stiffness, yield force, and maximum force were compared between groups by one-way ANOVA. *P*-values < 0.05 were considered significant for all hypothesis tests.

### Multi-screws stability of plate fixation test

To test the screw stability when being applied in a plate, polyurethane foam blocks were cut into small pieces of 30 × 30 × h60 mm to represent the proximal fragment of a long bone fracture. Afterwards, one end of the plate was secured to the foam block using two of the same types of screws, so as to build the fracture plate fixation construct. In this study, the fixation stabilities of locking screws and compression screws applied to a plate were tested, so LP and DCP were used with their respective screw types. Three groups were set for each plate fixation type, including plates fixed by buttress-, triangle-, and square-threaded screws, with 5 replicate models in each group. In the LP fixation model, the plate was offset by 2 mm from the bone [28]. In the DCP fixation model, a maximum torque of 1 Nm was utilized to tighten the compression screw with a torque control screwdriver [29, 30].

The end of the plate not attaching to the bone was fixed vertically on the load cell (0–1000 N) and later a loading force was applied onto the upper surface of the foam block using an MTS Mini Bionix system (Fig. 2 c and d). Craniocaudal loading and torsional loading are the two main loading types for the long bones of the arms and legs during daily activity. Therefore, they were adopted to test the fixation stability of the screws. Under cyclic craniocaudal loading, the force applied was started at 100–200 N and increased by 50 N every 100 cycles (Fig. 2 c). When the MTS detected a total craniocaudal displacement of 5 mm, the cyclic craniocaudal loading of the foam block was terminated. Then, the force and number of cycles required to achieve 1-, 2-, 3-, 4-, and 5-mm displacements were recorded for each screw type. For cyclic torsional loading, the torque applied was started at ±1 Nm and increased by 0.5 Nm every 100 cycles (Fig. 2 d). The cyclic torsional loading of the foam block was terminated once a total torsional angle of 10° was detected by MTS. Thus, the torque and number of cycles required to achieve 2, 4, 6, 8, and 10 degrees of torsion were recorded for each screw type. The differences in fixation stability among the three screw types were analyzed using one-way ANOVA. *P*-values < 0.05 were considered significant for all hypothesis tests.

## Results

### Individual screw axial pullout test

**Fig. 3:**
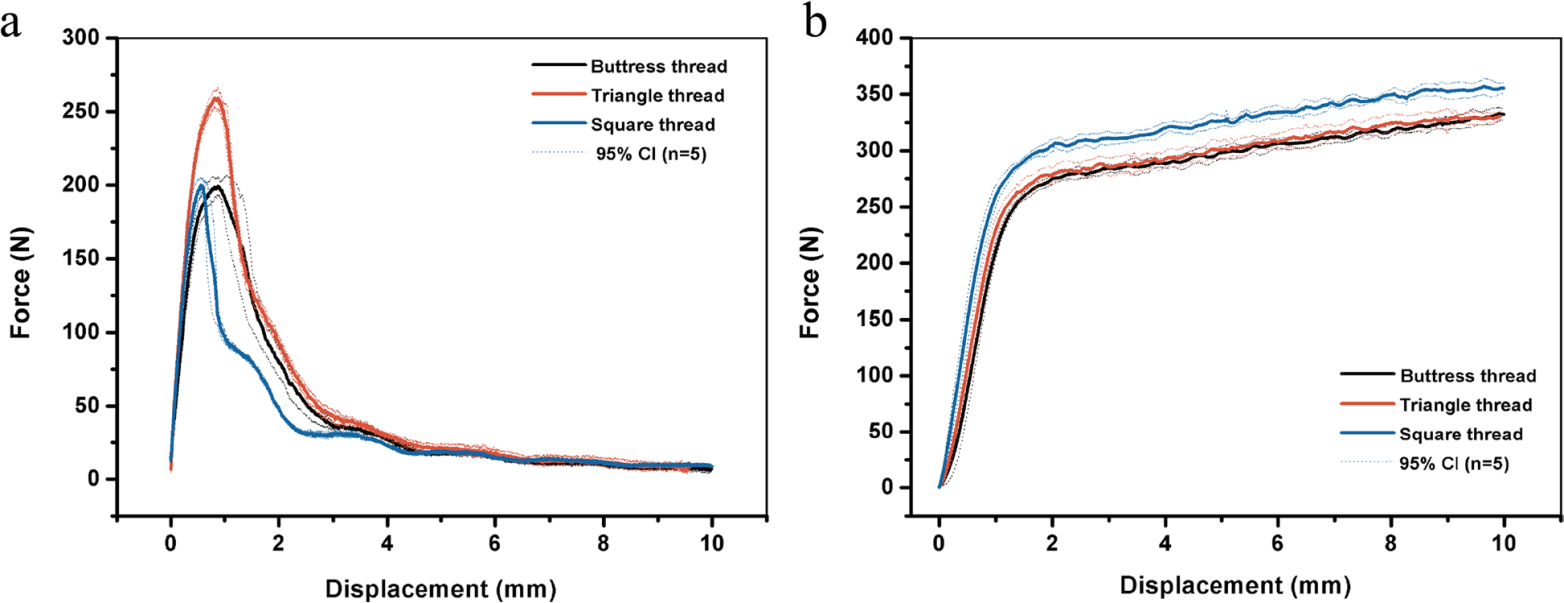
Mean force with 95% CI (N) vs. displacement (mm) curve for the buttress-, triangle-, and square-threaded screws during axial pullout (**a**) and lateral migration (**b**) through a foam block at 5 mm/min

The force-displacement curves in the axial pullout test are presented in Fig. 3 a. Apparently, the triangle-threaded screws demonstrated better performance than the square- and buttress-threaded screws subject to axial pullout. Table 1 displays the relevant parameters, including stiffness, maximal force and yield force. Obviously, square- and triangle-threaded screws had increased stiffness compared with buttress-threaded screws. Besides, triangle-threaded screws exhibited obviously stronger maximal force and yield force than squared- and buttress-threaded counterparts.

**Table 1:** Stiffness, yield force, and maximum pullout force for the buttress-threaded screw, triangle-threaded screw, and square-threaded screw during axial pullout (*n* = 5 for all groups)

	Stiffness Mean ± SD (N/mm)	Yield force Mean ± SD (N)	Max. pullout force Mean ± SD (N)
**Buttress thread**	373.62 ± 50.89	154.99 ± 9.63	195.16 ± 1.41
**Triangle thread**	556.81 ± 40.98	184.08 ± 7.50 N	256.25 ± 8.95
**Square thread**	489.29 ± 91.52	152.42 ± 12.35	191.33 ± 6.10
***p*-value (one-way ANOVA)**	*p*<0.05	*p*<0.05	*p*<0.05

### Lateral migration of individual screw test

Figure 3 b displays a curve of force as a function of displacement in the lateral migration test. It was observed that square-threaded screws demonstrated superior performance to buttress- and triangle-threaded screws. Table 2 presents relevant parameters, including stiffness, yield force, and maximum force. Clearly, square-threaded screws had obviously higher stiffness, stronger maximum force and yield force than buttress- and triangle-threaded screws.

**Table 2:** Stiffness, yield force and maximum force for the buttress-threaded screw, triangle-threaded screw and square-threaded screw during lateral migration (*n* = 5 for all groups)

	Stiffness Mean ± SD (N/mm)	Yield force Mean ± SD (N)	Force at 5 mm displacement Mean ± SD (N)
**Buttress thread**	263.29 ± 19.86	263.26 ± 5.20	298.22 ± 6.56
**Triangle thread**	253.35 ± 26.26	265.17 ± 6.49	300.78 ± 9.46
**Square thread**	325.66 ± 36.71	291.61 ± 5.58	326.03 ± 5.33
***p*-value (one-way ANOVA)**	*p*<0.05	*p*<0.05	*p*<0.05

### Multi-screws stability of DCP fixation test

**Fig. 4:**
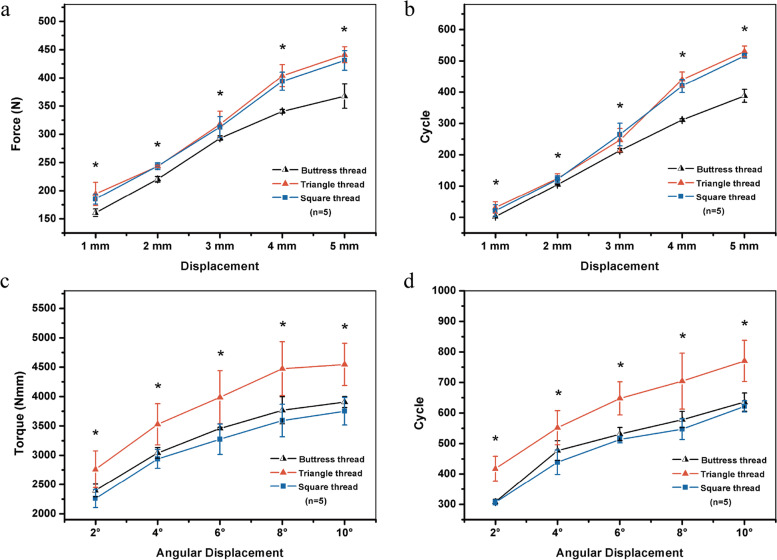
Fixation stabilities of buttress-, triangle- and square-threaded screw–DCP constructs implanted in the PU foam under cyclic craniocaudal loading (**a**, **b**) and cyclic torsional loading (**c**, **d**) of the foam block. (One-way ANOVA: *P ≤ 0.05)

In the DCP/compression screw constructs subject to craniocaudal loading, compared wiht buttress-threaded screws, both the squared- and triangle-threaded screws required more cycles and stronger force to achieve an identical displacement. For triangle- and square-threaded screws, the cycle number and load needed to obtain an identical displacement were similar, with no significant difference (Fig. 4 a and b).

In the DCP/compression screw constructs subject to torsional loading, compared with buttress- and square-threaded screws, a greater number of cycles and increased torque were needed for triangle-threaded screws to obtain an identical torsion angle. For buttress- and square-threaded screws, similar cycle number and torque were needed to achieve an identical displacement, and the difference was not significant (Fig. 4 c and d).

### Multi-screws stability of LP fixation test

**Fig. 5:**
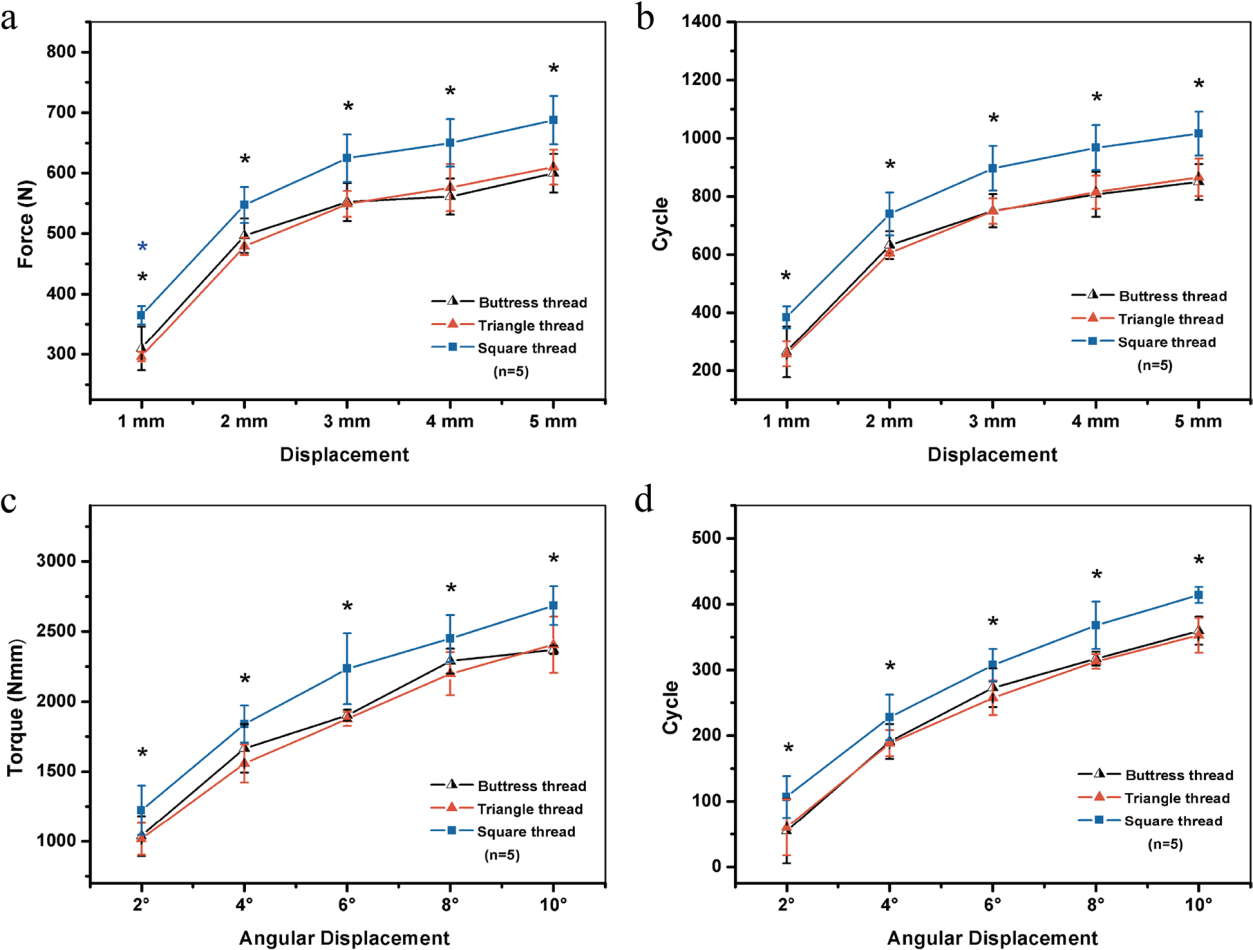
Fixation stabilities of buttress-, triangle-, and square-threaded screw–LP constructs implanted in the PU foam under cyclic craniocaudal loading (**a**, **b**) and cyclic torsional loading (**c**, **d**) of the foam block. (One-way ANOVA: *P ≤ 0.05)

In the LP/locking screw constructs subject to craniocaudal loading, a greater number of cycles and an increased load were required for the square-threaded locking screws to obtain the same displacement as those of buttress- and triangle-threaded screws. There were significant differences in the cycle number and load to achieve 1–5 mm displacements between screws of the two thread types. Buttress- and triangle-threaded screws required similar cycle number and force for obtaining an identical displacement, and there was no significant difference (Fig. 5 a and b).

In the LP/locking screw constructs subject to torsional loading, a greater number of cycles and a higher torque were needed for the square-threaded screws to acquire an identical torsion angle to those of buttress- and triangle-threaded screws. Differences in the cycle number and torque needed to obtain the torsion at 2/4/6/8/10 degrees were significant between the two thread types. The buttress- and triangle-threaded screws had similar requirements on cycle number and torque to obtain an identical displacement, with no significant difference (Fig. 5 c and d).

## Discussion

The individual screw tests revealed that screws with triangle threads were superior in axial pullout strength, while those with square thread were superior in lateral migration resistance, and those with buttress thread were inferior in both axial pullout strength and lateral migration resistance. The buttress thread was proposed by Robert Danis in the 1940s as the standard thread for bone screws, which did not perform well in the axial pullout test. The triangle thread, which was the standard thread of the industrial screw for connection, however, had the best axial pullout strength. In this study, the pullout strength of the triangle-threaded screw was 31.3% higher than that of the buttress-threaded screw. This was consistent with the finding by Kim et al. in a pedicle screw-related biomechanical test that the strangle thread had the highest pullout strength compared with buttress thread and square thread [17]. This indicates that for bone screws that are mainly subject to pullout force or rely on the pullout strength to exert its functions, such as lag screws and anchor screws, a triangle thread can greatly improve the fixation strength. Several finite element analysis (FEA) studies have been performed to investigate the effect of thread profiles on the stress concentration and distribution in the surrounding bone tissues. For instance, Geng et al. found that triangle thread and the broader square thread generated significantly less stress than the thinner and narrower square thread in the cancellous bone [31]. Erslan et al. proved that the maximum von Mises stress value of the square thread was the lowest compared with triangle thread, buttress thread, and reverse buttress thread [32]. However, Oswal et al. discovered that the reverse buttress thread had less stress concentration than triangle thread and buttress thread [33]. Such inconsistent results found in the FEA can be ascribed to the different testing setups and loading conditions among different studies. For the lateral migration test, the square-threaded screws performed best, with an improvement of approximately 10% compared with the buttress- and triangle-threaded screws. This may explain why some medical device companies manufacture pedicle screw products with square thread rather than triangle or buttress threads.

Later, this study compared the screws applied to a DCP in pairs under cyclic craniocaudal loading, as a result, the square-threaded screws had comparable fixation stability to triangle-threaded screws, and both of them had better fixation stabilities than buttress-threaded screws. Under the cyclic torsional loading, screws with triangle threads had better fixation stability than those with buttress and square threads, and those with buttress thread achieved comparable fixation stability to those with square thread. These results indicate that when standard buttress-threaded screws are used as the compression screws to repair fractures in conjunction with a DCP, they have the worst fixation stability under both craniocaudal and torsional loadings. The triangle thread, however, displayed better performance. This suggests that a compression screw product should have a triangle thread design, rather than the standard buttress thread design, so as to improve its fixation strength.

For the locking screws applied to a LP in pairs, screws with square thread had better fixation stability than those with buttress and triangle threads, and those with buttress thread attained comparable fixation stability to those with triangle thread under both craniocaudal and torsional loadings. These results reveal that when being used as the locking screws to repair fractures in conjunction with a LP, the standard buttress thread and triangle thread demonstrate the worst fixation stability under both craniocaudal and torsional loadings. The square thread, however, had better performance. This demonstrates that a locking screw product should have a square thread design, rather than the standard buttress or triangle thread design, for the sake of improving its fixation strength. However, the area of the square thread profile is twice of that of buttress and triangle threads, which suggests that more bones will be damaged and removed, and a higher insertion torque will be required when the screw is inserted. This may limit its application in bone screw design.

Thread is an important structure for bone screws when designing devices that will be subject to multiple loading conditions. Different thread shapes allow for modifications in the contact surface area and shear force development at the screw-bone interface, thereby affecting the fixation stability of the device. Previously, the fixation stability of bone screws has been consistently evaluated by a pure axial pullout test [34–37]. It is well known that the application of pullout tests in the axial direction is not clinically realistic, since it rarely simulates most of the physiological loading conditions [38, 39]. Nonetheless, the axial pullout test is the exclusive standardized method to test the anchorage strength of medical bone screws [19]. In this study, in addition to the axial pullout test, the lateral migration test, which is more relevant to the physiological loading conditions [18, 19], was also performed in the individual screw biomechanical test. The fixation stabilities of the screws applied to a LP and a DCP in pairs under cyclic craniocaudal loading and torsional loading were also evaluated and compared. In this study, a synthetic homogeneous material was used as a support to prevent any interference by uncontrollable variables of the cadaveric bones. The selection of foam bone allowed us to recreate the worst possible clinical situation where the bone could not hold the screws firmly under in vitro conditions, so as to better evaluate the fixation stabilities of different screw designs.

The buttress thread, triangle thread, and square thread tested in this study were all from the industrial screw thread designs. None of them demonstrated superior performance in all the individual screw biomechanical tests and multi-screws biomechanical tests in our study. Moreover, the influences of these three different thread designs on the insertion torque of the bone screw remain unclear. Screw threads designed specifically for human-related physiological loading with modest insertion torque should be developed, instead of directly relying on the existing thread designs from the industrial screws.

Several limitations of the present study should be considered. First, synthetic bone models are a consistent medium for mechanical testing, which do not perfectly simulate normal human bone. However, the polyurethane foam is microstructurally similar to human bone and exhibits similar mechanical properties while avoiding the inconsistencies induced by interindividual variations in human bones. Second, since the bone samples used in this study were synthetic cancellous bones, which were unable to represent the clinical environment in which the cortical bone was the main factor of bone screw stability. Thus, more research is needed to evaluate how the three different thread types perform in a bone analog with cortical shell. Third, the present work utilized torsional loading and cyclic craniocaudal loading to simulate physiological loading, while these two loading types did not comprehensively mimic the extremely complicated arm or leg loading conditions in human body during daily activities. Nonetheless, the loadings utilized in the present work mostly simulated our daily activities. At last, the present biomechanical research analyzed the simulated bone under normal density condition as an example. In the future, more studies should be conducted to test bone densities that represent osteoporosis and osteopenia to strengthen our conclusion. The conclusions of this study were drawn taking these limitations into account.

## Conclusions

In summary, the triangle-threaded screws had superior pullout strength, while square-threaded screws demonstrated the highest lateral migration resistance. The DCP fixation stability under craniocaudal loading was not different between triangle- and square-threaded screws, while both of them had superior fixation stabilities to buttress-threaded screws. Under torsional loading, DCP fixation with triangle-threaded screws achieved superior fixation stability. LPs with square-threaded screws required a larger force and more cycles to reach the same amount of displacement under both craniocaudal loading and torsional loading. The findings of this study may have limited clinical significance, but they will provide new insight for surgeons and bone-implant device companies and will eventually benefit clinical practice.

## Data Availability

All data generated or analysed during this study are included in this published article.

## Abbreviations

DCP: Dynamic compression plate
LP: Locking plate
FEA: Finite element analysis
